# Global/integral assays in hemostasis diagnostics: promises, successes, problems and prospects

**DOI:** 10.1186/s12959-014-0032-y

**Published:** 2015-01-28

**Authors:** Mikhail A Panteleev, Hendrik Coenraad Hemker

**Affiliations:** Center for Theoretical Problems of Physicochemical Pharmacology, Moscow, Russia; Federal Research and Clinical Center of Pediatric Hematology, Oncology and Immunology, Moscow, Russia; Faculty of Physics, M.V. Lomonosov Moscow State University, Moscow, Russia; Synapse BV, CARIM, Maastricht University, Maastricht, the Netherlands

Laboratory testing in hemostasis is a subject of growing concern among the specialists. To put it plainly, the standard assays that have been in widespread use for more than 40 years (APTT and PT assays for clotting, aggregometry for platelets) are not sensitive and specific for too many major disorders of hemostasis; their parameters can remain within normal ranges when patient is in acute danger of thrombosis or bleeding; even if they do detect some abnormality they do not indicate the severity of the clinical manifestations; in rare cases they are even abnormal in a clinically asymptomatic individual.

Admittedly, as already recognized by Virchow in his famous Virchow’s triad, thrombosis can arise from disorders in either vessel wall, blood flow, or blood composition and laboratory diagnostics only concerns the latter. If the origin of a thrombotic or bleeding disorder is not in the blood, as in myocardial infarction caused by atherosclerotic plaque rupture, there is no need for the blood to be in a hyper-coagulable state before the event and laboratory diagnostics of coagulation or platelet function can be completely normal (although some non-hemostatic biomarkers for e.g. atherosclerotic plaque instability do exist [1]).

The same holds for hemostasis: the extent of bleeding is determined by the state of the tissue (type and degree of damage, tissue composition at the injury site), physical parameters of the wound and blood flow (geometry of the wound, blood flow patterns there) as well as blood biochemistry. No functional assay of blood clotting is likely to be able to predict hemorragic stroke resulting from aneurism rupture or bleeding in a person with hereditary hemorrhagic telangiectasia or Ehlers–Danlos syndrome, exactly because there is originally nothing wrong with their blood in these cases.

Nevertheless, even in a perfectly normal population the function of the clotting system does determine the likelihood and degree of hemostasis and thrombosis. It is for instance well documented that normal persons with blood group O bleed more and have a smaller risk of thrombosis than those with non-O blood groups [2-5]. This is directly related to their having less factor VIII and Von Willebrand factor and higher anti-thrombin activity.

Recognizing that the “coagulability” of the blood tells only part of the story cannot justify to acquiesce to the inefficiency of the traditional clotting- and platelet assays. There are too many cases where their results are not correct, even when the origin of the hemostatic abnormality is clearly systemic and is to be found in the blood. A typical example is *the hypercoaguable state* that results from the presence of circulating coagulation activators in blood (microparticles activating clotting via tissue factor [6] or contact pathway [7], long-lived clotting enzymes like factors IXa, XIa, and XIIa [7-9]). One of the leading Russian hematologists, Andrey Vorobyov, recommended spontaneous clotting of blood in the syringe needle observed during blood collection as one of the typical indicators of the hypercoagulative stage of disseminated intravascular coagulation, usually coinciding with normal APTT [10], and cited this as a regrettable example of why a doctor can hardly rely on the available traditional laboratory diagnostics tools in coagulation.

Less striking examples include the lack of correlation between prolongation of APTT and clinical manifestations in all hemophilias and factor XII deficiency; the insensitivity of clotting assays to thrombophilias like factor V Leiden; the insensitivity of platelet aggregometry to many platelet disorders like Scott syndrome and gray platelet syndrome and its inapplicability in thrombocytopenia, etc. Then there is the problem of the control of anticoagulant therapy. A profound international effort has succeeded to establish the INR as a reliable way to control anti-vitamin K prophylaxis. For heparins, however, the APTT is worthless but for the detection of over-dosage and the common believe that low molecular weight heparins should be administered in standard dosages only hides our incapacity to establish their effect in a patient with any degree of accuracy. This is the more serious as there are large individual differences in the response to the same dose of heparin [11].

A particulary severe problem is monitoring of the novel pro-coagulant and anti-thrombotic agents: traditional clotting methods (for coagulation) or aggregometry (for platelets) either do not detect effects of recombinant activated factor VII, anti-TFPI agents, new direct anticoagulants, P2Y12 antagonists or do this without clear relation to clinical efficiency.

Why is it so? Is there a way to resolve this inefficiency of traditional assays?

A reasonable explanation is that these assays do not meet the criteria of an adequate physiological function test: if circulating microparticles, or factor XIIa, or the factor V_Leiden_ mutation increase thrombotic risk but do not affect APTT then there is something wrong with the APTT design. The two possible solutions are obvious: a) to develop adequate physiological function tests that reflect all complexities of in vivo clotting or (inclusive or!) b) to complement them by molecular diagnostic tools able to sense the individual (risk) factors.

The second approach, despite being less decisive and somewhat ambiguous, has become state of the art in the last decennia, is intuitively simpler and is being thoroughly explored and widely used. At the present moment, a doctor has in its arsenal a wide range of methods to determine levels of individual clotting factor precursors, inhibitors, active circulating factors, microparticles of various origin, pro-thrombotic mutations, anti-Xa activity, fibrinolysis activity, expression of various proteins on the platelet membrane, platelet granule contents, and many others. This ‘coagulogram’ is important, because in many cases it allows determination of the specific mechanisms behind the thrombotic or bleeding disorder. Still, this approach has major limitations: a) the large and extremely expensive series of possible tests is far from being complete and cannot become comprehensive, the blood coagulation network and platelets being as complex as it is; b) knowledge about the individual components does not allow to estimate the over-all function, will not give a conclusive estimate of the overall hemostatic/thrombotic risk. Thrombotic or bleeding risk results from a combination of changes in several of the components of the hemostatic system [12]. As in the case of the O and non-O blood groups, all individual factors may be in the normal range and yet the hemostatic/thrombotic tendency is significantly influenced. In liver cirrhosis the lack of prothrombin is compensated for by a lack of antithrombin, that itself is partly compensated by a rise in α2-macroglobulin (Kremers RMW, Kleinegris M-C, Cate HT, Wagenvoord RJ, Hemker HC: Decreased prothrombin conversion and thrombin inactivation result in rebalanced thrombin generation in liver cirrhosis. In preparation). Sometimes platelets can even compensate for the defects of plasma coagulation [13].

There should be a way to evaluate the overall interaction for diagnostic reasons (is the problem to be sought in the blood?), for screening of hemostatic risks (e.g. preoperatively), for screening of thrombotic risk (is prophylaxis indicated?) and for measuring the effects of prophylactic- and therapeutic measures. In short, there should be global-, integral assays of hemostasis [14,15].

It is hard to give a precise definition of “global assay”. It could be said that they are function tests of the hemostatic system, where assay conditions are chosen so that the measured parameters reflect the interaction of all components of the hemostatic system in the same way as they do in vivo; thus allowing adequate evaluation of their contribution and interaction with each other. Indeed, global assays are experimental in vitro models of hemostasis/thrombosis that aim to mimic all important aspects of the physiological (and pathological) system both as to biochemistry and biophysics. Complete “physiological adequacy” is the holy grail of the ultimate function testing but also partly solutions are already welcome.

Complete physiological adequacy may be asking too much. None of the existing global assays actually involves all components of hemostasis. The thrombin generation (TG) assay, one of the leading and established methods of this class, focuses on blood coagulation, though it can detect platelet functions via their effect on blood coagulation [16]. Within blood coagulation, use of thrombin generation assay for diagnostics of defects in fibrinogen, fibrinolysis, protein C system usually requires some modifications of the assay [17]. Thromboelastography, another major global approach, is sensitive for defects in platelet aggregation and fibrinolysis, but not platelet adhesion. Clot waveform analysis is actually an advanced version of APTT, with critically important advantages but with many of its limitations remaining in place. Thrombodynamics explicitly includes spatial heterogeneity of blood coagulation, spatial propagation and diffusion of clotting enzymes, but not platelet aggregation or adhesion [7]. Various perfusion flow systems for observation of thrombus formation in vitro aim to add platelet adhesion to the list, sometimes in combination with coagulation (two such systems currently available commercially are PFA and T-TAS [18]), but introduction of all this complexity raises completely new questions and issues.

The purpose of the paper collection “Global assays of hemostasis” published by the Thrombosis Journal that we present here is to summarize state-of-the-art knowledge in order to clearly describe the existing assays, their advantages and drawbacks, the possibilities to use them in everyday practice and problems to overcome [19-22].

Two papers in our collection deal with the description of the existing assays and those under development. The one by Lance focuses on the three established major assays: thrombin generation, thrombelastography and clot waveform analysis [20]. Another, by Tynngard et al., picks up the storyline to make a transition to description and critical analysis of new global assays ranging from free oscillation rheometry and thrombodynamics to flow perfusion chambers and PFA-100 [22].

Two other papers focus on the representative critical problems in the field. The contribution of Brinkman deals with the important aspect of working with novel oral anticoagulants [19]. One advantage of the global assays is that they (if physiologically adequate) can be used for the detection and monitoring of novel drugs that have no adequate effect on traditional assays (novel anticoagulants, bypassing agents, etc.). Indeed the rise of innovative oral anticoagulants is a major challenge for laboratory diagnostics these days, and one where global assays can be invaluable. Finally, the article by Lipets and Ataullakhanov investigates the above-described problem of hypercoagulation, insensitivity to which is also the major drawback of the traditional assays [21]. Their analysis aims to summarize the existing experience of employing global assays for the prediction of thrombotic risks of various origins.

Some of the issues remained outside the scope of the above discussion. One prominent problem is preanalytics: the high sensitivity of global assays makes them particularly vulnerable to inaccuracies and variables of sample collection and preparation [23,24]. It is said that Armand Quick calibrated his stopwatches on the length of the “prothrombin time” being twelve seconds. The robustness of the test alas reflects its insensitivity and the inverse is – regretfully – true as well, hence the problem of overall standardization of the assays [25].

A third problem is their mathematical modeling, its potential usefulness [26] and issues [27] (although presently the thrombin generation assay is the only well-established global assay, for which kinetic models do exist and that date from long before the present upsurge of the assay [28]). Finally, there remain many specific clinical problems apart from those described above and use of global assays for each of them deserves a separate discussion. A traditional special issue, like the present one cannot be expected to give a complete survey of all open problems.

Despite the promise and numerous successes the global assays still remain a diverse group of methods. In fact, when blood or plasma clots, there is hardly a physical parameter that does not change: turbidity, electrical conductance, viscocity, mechanical resistance, probably also NMR and so on and so forth. Each of them can be transformed into a global essay. The normal range of its parameters can be determined. Then we can establish the *correlation* between abnormal parameters and pathology and this may serve diagnostic purposes – may be called a global test A good function test, however, shows *correlation* because there is a *direct relation* between the outcome of the test and the (patho-) physiological function. Heart failure may be estimated from the degree of shortness of breath, nocturia, edema, venous pressure and a number of other signs and symptoms. Left ventricular function is at the basis of these phenomena and therefore that is the essential variable. In the same manner it appears to us that the generation of thrombin is the essential contribution of the plasma to hemostasis, a phenomenon that more directly than clotting times and other derived phenomena reflects an essential function.

The last word in these matters is to medical usefulness. Global methods are an advanced tool that still has a long way to go before becoming universally accepted in clinical practice.

It is our hope that the present volume can serve to make these assays more familiar to the hemostasis specialists and may contribute to their further development and spreading.

## References

[1] Shah PK (2014). Biomarkers of plaque instability. Curr Cardiol Rep.

[2] Gandara E, Kovacs MJ, Kahn SR, Wells PS, Anderson DA, Chagnon I (2013). Non-OO blood type influences the risk of recurrent venous thromboembolism. A cohort study. Thromb Haemost.

[3] O’Donnell J, Boulton FE, Laffan MA (2002). The relationship between plasma concentration of alpha2-macroglobulin and ABO blood group. Thromb Haemost.

[4] Dentali F, Sironi AP, Ageno W, Bonfanti C, Crestani S, Frattini F (2013). Relationship between ABO blood group and hemorrhage: a systematic literature review and meta-analysis. Semin Thromb Hemost.

[5] Meade TW, Cooper JA, Stirling Y, Howarth DJ, Ruddock V, Miller GJ (1994). Factor VIII, ABO blood group and the incidence of ischaemic heart disease. Br J Haematol.

[6] Geddings JE, Mackman N (2013). Tumor-derived tissue factor-positive microparticles and venous thrombosis in cancer patients. Blood.

[7] Lipets E, Vlasova O, Urnova E, Margolin O, Soloveva A, Ostapushchenko O (2014). Circulating contact-pathway-activating microparticles together with factors IXa and XIa induce spontaneous clotting in plasma of hematology and cardiologic patients. PLoS One.

[8] Minnema MC, Peters RJ, de Winter R, Lubbers YP, Barzegar S, Bauer KA (2000). Activation of clotting factors XI and IX in patients with acute myocardial infarction. Arterioscler Thromb Vasc Biol.

[9] Kohler HP, Carter AM, Stickland MH, Grant PJ (1998). Levels of activated FXII in survivors of myocardial infarction–association with circulating risk factors and extent of coronary artery disease. Thromb Haemost.

[10] Panteleev MA, Vasil’ev SA, Sinauridze EI, Vorobyev AI, Ataullakhanov FI. Practical Coagulation Science [in Russian]. Moscow; 2010.

[11] Al Dieri R, Alban S, Beguin S, Hemker HC (2006). Fixed dosage of low-molecular-weight heparins causes large individual variation in coagulability, only partly correlated to body weight. J Thromb Haemost.

[12] Danforth CM, Orfeo T, Everse SJ, Mann KG, Brummel-Ziedins KE (2012). Defining the boundaries of normal thrombin generation: investigations into hemostasis. PLoS One.

[13] Tarandovskiy ID, Balandina AN, Kopylov KG, Konyashina NI, Kumskova MA, Panteleev MA (2013). Investigation of the phenotype heterogeneity in severe hemophilia A using thromboelastography, thrombin generation, and thrombodynamics. Thromb Res.

[14] Brummel-Ziedins KE, Wolberg AS (2014). Global assays of hemostasis. Curr Opin Hematol.

[15] van Geffen M, van Heerde WL (2012). Global haemostasis assays, from bench to bedside. Thromb Res.

[16] Hemker HC, Al Dieri R, Beguin S (2004). Thrombin generation assays: accruing clinical relevance. Curr Opin Hematol.

[17] Eilertsen AL, Liestol S, Mowinckel MC, Hemker HC, Sandset PM (2007). Differential impact of conventional and low-dose oral hormone therapy (HT), tibolone and raloxifene on functionality of the activated protein C system. Thromb Haemost.

[18] Yamaguchi Y, Moriki T, Igari A, Matsubara Y, Ohnishi T, Hosokawa K (2013). Studies of a microchip flow-chamber system to characterize whole blood thrombogenicity in healthy individuals. Thromb Res.

[19] Brinkman HJM. Global assays and the management of oral anticoagulation. Thromb J. 2014, This collection: in press. 10.1186/s12959-015-0037-1 PMC4355453 25762867

[20] Lance M. A general review of major global coagulation assays: thrombelastography, thrombin generation test and clot waveform analysis. Thromb J. 2014, This collection: in press. 10.1186/1477-9560-13-1 PMC4417204 25937820

[21] Lipets EN, Ataullakhanov FI. Global assays of hemostasis in the diagnostics of coagulation and evaluation of thrombosis risks. Thromb J*.* 2014, This collection: in press. 10.1186/s12959-015-0038-0 PMC4310199 25635172

[22] Tynngard N, Lindahl TL, Ramstrom S. Assays of different aspects of hemostasis – what do they measure? Thromb J. 2014, This collection: in press. 10.1186/s12959-015-0036-2 PMC4329663 25688179

[23] Dashkevich NM, Vuimo TA, Ovsepyan RA, Surov SS, Soshitova NP, Panteleev MA (2014). Effect of pre-analytical conditions on the thrombodynamics assay. Thromb Res.

[24] Curvers J, Christella M, Thomassen LG, de Ronde H, Bertina RM, Rosendaal FR (2002). Effects of (pre-)analytical variables on activated protein C resistance determined via a thrombin generation-based assay. Thromb Haemost.

[25] Dargaud Y, Wolberg AS, Luddington R, Regnault V, Spronk H, Baglin T (2012). Evaluation of a standardized protocol for thrombin generation measurement using the calibrated automated thrombogram: an international multicentre study. Thromb Res.

[26] Brummel-Ziedins KE, Orfeo T, Rosendaal FR, Undas A, Rivard GE, Butenas S (2009). Empirical and theoretical phenotypic discrimination. J Thromb Haemost.

[27] Hemker HC, Kerdelo S, Kremers RM (2012). Is there value in kinetic modeling of thrombin generation? No (unless…). J Thromb Haemost.

[28] Willems GM, Lindhout T, Hermens WT, Hemker HC (1991). Simulation model for thrombin generation in plasma. Haemostasis.

